# Passive torque influences the Hoffmann reflex pathway during the loading and unloading phases of plantar flexor muscles stretching

**DOI:** 10.14814/phy2.14834

**Published:** 2021-05-01

**Authors:** Mehdi Datoussaid, Hasnae El Khalouqi, Charel Dahm, Nathalie Guissard, Stéphane Baudry

**Affiliations:** ^1^ Laboratory of Applied Biology & Research Unit in Applied Neurophysiology (LABNeuro) ULB‐Neurosciences Institute (UNI) Université Libre de Bruxelles (ULB) Brussels Belgium

**Keywords:** Golgi tendon organ, homosynaptic depression, Ib afferents, muscle spindle, ultrasound

## Abstract

This study investigated the influence of passive tension on Hoffmann reflex during the loading (muscle stretched by passive joint movement) and unloading phase (joint returned to initial position) of muscle stretching. The maximal H‐reflex amplitude (*H*_max_) was recorded in soleus in 19 young adults during the loading and unloading phases of a passive 30° dorsiflexion, from 90° ankle angle (reference position). *H*_max_ was evoked at similar angles (protocol‐1) or similar passive torque (PT; protocol‐2) during the loading and unloading phases, or during two loading phases separated by a 5‐min stretch hold at 30° ankle dorsiflexion relative to the reference position (protocol‐3). Homosynaptic depression (HD) was assessed with paired H reflexes (0.5‐s interstimulus interval) during the loading and unloading phases (protocol‐4; *n*=13). In protocol‐1, PT was lesser and *H*_max_ greater during the unloading than the loading phase (*p* < 0.001). In protocol‐2, no difference in *H*_max_ was observed between phases. In protocol‐3, PT was lesser and *H*_max_ greater during the second than the first loading phase (*p* < 0.001). Changes in PT during in these three protocols were associated with those in *H*_max_ (*r* ^2^ ≥ 0.97). In protocol‐4, HD increased and decreased during the loading and unloading phases, respectively (*p* < 0.001), without differing between phases. Additional experiments (*n*=12) showed a similar modulation of *H*_max_ in gastrocnemius medialis during loading and unloading phases, while muscle fascicle length did not differ between phases. This study indicates that the H‐reflex modulation during muscle stretching relies in part on mechanisms associated with the PT developed by the muscle‐tendon unit.

## Graphical abstract

Hypothetical mechanisms involved in the increase in the responsiveness of the H‐reflex pathway with the decrease in passive tension of the muscle‐tendon unit in response to acute stretching (PAD: primary afferent depolarisation interneurons).

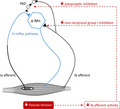

## 1 INTRODUCTION

Muscle stretching is commonly performed in sport and rehabilitation (Behm et al., 2016) to increase the extensibility of the muscle–tendon unit (MTU) and joint range of motion (Kawakami et al., 2008). When the MTU is forcibly stretched by passive joint mobilization (loading phase), the increase in passive torque (PT) largely reflects the stiffness of the MTU (Kawakami et al., 2008; Riemann et al., 2001). During the unloading phase (when the joint returns to its initial position), PT decreases but is less than during the loading phase for similar joint angles—that is, *elastic hysteresis* (Giuliani et al., 2019; King et al., 2016; Morse et al., 2008; Nordez et al., 2009; Taylor et al., 1990). The elastic hysteresis corresponds to the difference between the loading and unloading curves in a strain–stress cycle due to material internal friction (energy loss in the form of heat). The viscoelastic properties of the MTU are also characterized by the stress‐relaxation phenomenon (Duong et al., 2001; Kato et al., 2011), which corresponds to the torque decay at a constant MTU length (Lim et al., 2019; Maganaris & Paul, 2000). Stress relaxation can be quantified as the reduction in PT during a static stretching (Kato et al., 2011).

Another common observation during passive stretching of the ankle plantar flexors MTU is the reduction in the amplitude of the Hoffmann (H) reflex in the soleus (SOL) muscle (Guissard et al., 1988; Pinniger et al., 2001), which reflects pre‐ and post‐synaptic inhibitory processes (Guissard & Duchateau, 2006). It is worth noting that during the loading phase, the greater the stretching intensity (extent of MTU lengthening), the greater the PT and the lesser the H‐reflex amplitude (Guissard et al., 1988). This can partly highlight the influence of passive tension on H‐reflex pathway through the non‐reciprocal group I inhibition originating from Golgi tendon organ (Guissard & Duchateau, 2006). A similar mechanism should occur during the unloading phase of a stretch maneuver due to the elastic hysteresis. However, no data are currently available on the modulation of the H‐reflex pathway during the unloading phase. Compelling data on the relation between the H reflex and PT should enable a better understanding of the influence of PT on neural modulation during stretching maneuvers, and more generally on the interactions between muscle mechanics and neural modulation.

Therefore, our study investigated the influence of PT on SOL H‐reflex amplitude during passive stretching of the ankle plantar flexor muscles. We focused on the loading and loading phases as they are associated with large changes in PT and H‐reflex amplitude. These phases should thereby be beneficial for the study of the relationship between the PT and H reflex pathways. To this end, a main experiment consisting of four distinct protocols was designed. In protocol 1, H reflexes were recorded in SOL at similar ankle angles during the loading and unloading phases of a stretching maneuver, with the hypothesis of a greater H‐reflex amplitude during the unloading phase compared with the loading phase due to the elastic hysteresis. In protocol 2, H reflexes were evoked during the unloading phase at ankle angles adjusted to match the PT recorded during the loading phase. We hypothesized a similar H‐reflex amplitude during the loading and unloading phases due to the absence of a difference in PT between the two phases. In protocol 3, H reflexes were evoked during two loading phases separated by a 5‐min static stretching aimed at reducing PT during the second relative to the first loading phase. We hypothesized a greater amplitude of the H reflex during the second loading phase in response to the lesser PT. In protocol 4, we investigated the modulation of homosynaptic depression (HD), which corresponds to a decrease in neurotransmitter release by Ia afferent terminals during repetitive activation (Hultborn et al., 1996). A decrease in HD has been suggested as a potential mechanism to account for the increase in H‐reflex amplitude during (Budini et al., 2018) and immediately after (Budini et al., 2017) MTU stretching. Accordingly, we hypothesised HD to be lesser during the unloading than the loading phase. Finally, an additional experiment investigated whether the difference in H‐reflex amplitude between loading and unloading phases results from differences in muscle fascicle length between phases. Together, these protocols provide original and relevant data to document the influence of PT on the modulation of the H‐reflex pathway during passive stretching.

## 2 METHODS

### 2.1 Participants

Nineteen young adults (aged 24 [2] years; mean [standard deviation, SD], 6 women), free of any neurological or orthopedic damage of the lower limb, volunteered to visit the laboratory on two separate occasions to perform three experimental protocols. Of these participants, 13 (24 [2] years, 2 women) returned to the laboratory to perform a fourth experimental protocol. In addition, 12 participants who did not participate to the main experiment volunteered to engage in an additional experiment (23 [2] years, 6 women). Each participant was asked to not consume caffeine at least 6 h prior to their experimental session, and to refrain from intense exercise for 72 h before each experimental session. Approval for the project was obtained from the ULB‐Erasme Ethics Committee, and all procedures implemented in this study are in accordance to the Declaration of Helsinki.

### 2.2 Ergometric apparatus

The experiment was performed on a specific ergometer consisting of a pedal system fixed on a table (Abellaneda et al., 2009). The pedal was connected with a steel cable to a mechanical device that enabled graduated passive dorsiflexion of the ankle. A strain‐gauge transducer (Type 4576A2NC1, Kistler, CH) was placed between the steel cable and the mechanical device to record PT during the stretching maneuvers. The angular displacement of the pedal was measured from the signal of an electronic inclinometer (Type PTAM27, Binz Technics, BE). During the experimental sessions, participants laid prone on the table with both legs extended and the foot of the dominant leg secured by three straps to the pedal. The first strap was placed over the dorsum of the foot, and two other straps were attached in opposite directions around the ankle.

### 2.3 EMG recordings

Surface electromyography (EMG) was recorded from the SOL muscle of the dominant leg (identified as the one used to kick a ball) with surface electrodes (silver–silver chloride electrodes with 8 mm diameter) placed in a bipolar configuration with an interelectrode (center to center) distance of 2 cm. Before attaching the electrodes, the skin was shaved (when necessary) and cleaned with a solution of alcohol, ether, and acetone to reduce the impedance at the skin‐electrode interface. The electrodes were filled with gel and attached with adhesive tape, 3 cm below the muscle–tendon junction of the gastrocnemius medialis (GM) muscle, in line with the Achilles tendon. The reference electrode was placed over the tibia. The EMG signals were amplified (1000×) and band‐pass filtered (10–1000 Hz) with a custom‐made amplifier, prior to A/D sampling at 2 kHz (Power 1401, 16‐bit resolution, Cambridge Electronic Design) and stored on a computer.

### 2.4 Electrical stimulation

Electrical stimuli (1‐ms duration) applied to the tibial nerve were delivered via a constant current stimulator (DS7A, Digitimer), which was connected to surface electrodes (silver–silver chloride electrodes with 8 mm diameter) attached to the skin of the dominant leg with adhesive tape: the cathode was placed in the popliteal fossa and the anode was located just above the patella. The optimal site of stimulation was determined by moving the cathode until the site to elicit an H reflex in SOL with the largest amplitude at a given intensity was identified. The input–output relations for the H reflex and M wave were determined, at an ankle angle of 90°, by progressively increasing the current in steps of 0.5–1 mA (five stimulations/step) until the M‐wave amplitude reached a plateau (*M*_max_). From the input–output curves, the current intensity associated with the maximal amplitude of the H reflex (*H*_max_; protocols 1, 2, and 3, and Additional Experiment) or with an H‐reflex amplitude of 50% of *H*_max_ (*H* _50_; protocol 4), and the intensity corresponding to 1.2x *M*_max_ intensity (protocols 1, 2, 3, and 4, and Additional Experiment) were determined. The *H*_max_ was used in protocols 1, 2, and 3 to ensure its recording although a possible drastic decrease during the stretching maneuver, as reported previously (Guissard et al., 1988; Pinniger et al., 2001). Although determined as maximal in one situation, the amplitude of *H*_max_, which reflects excitatory and inhibitory processes at the spinal level, can decrease or increase depending on background muscle contraction and joint angle (Frigon et al., 2007). The use of *H*_max_, therefore, did not impede the possibility to record an upward or downward modulation during the stretching maneuver. The *H* _50_ used in protocol 4 was chosen to allow for the adjustment of the current intensity during the loading and unloading phases to obtain similar *H*_1_ amplitudes at each ankle angle in both phases; a constant amplitude of *H*_1_ is a crucial factor when evaluating HD (see below) (Takahashi et al., 2013).

### 2.5 Main experiment

In all protocols, the plantar flexor muscles of the dominant leg were stretched by mobilizing the ankle in 10° steps (1°/s) from the reference position (90° ankle angle—as measured as the angle between the sole of the foot and the axis passing through the head of the fibula and the lateral malleolus—referred hereafter as 0°) to 30° of ankle angle (dorsiflexed ankle joint position), which corresponded to 95%–100% of the maximal range of motion for all participants. Each ankle angle during the loading and unloading phase (10°, 20°, and 30°) was maintained for 30 s to allow the recording of the evoked potentials: five *H*_max_, evoked 5 s apart, and one *M*_max_ (protocols 1, 2, 3), or four pairs of H reflexes and one *M*_max_ (protocol 4).

**FIGURE 1:**
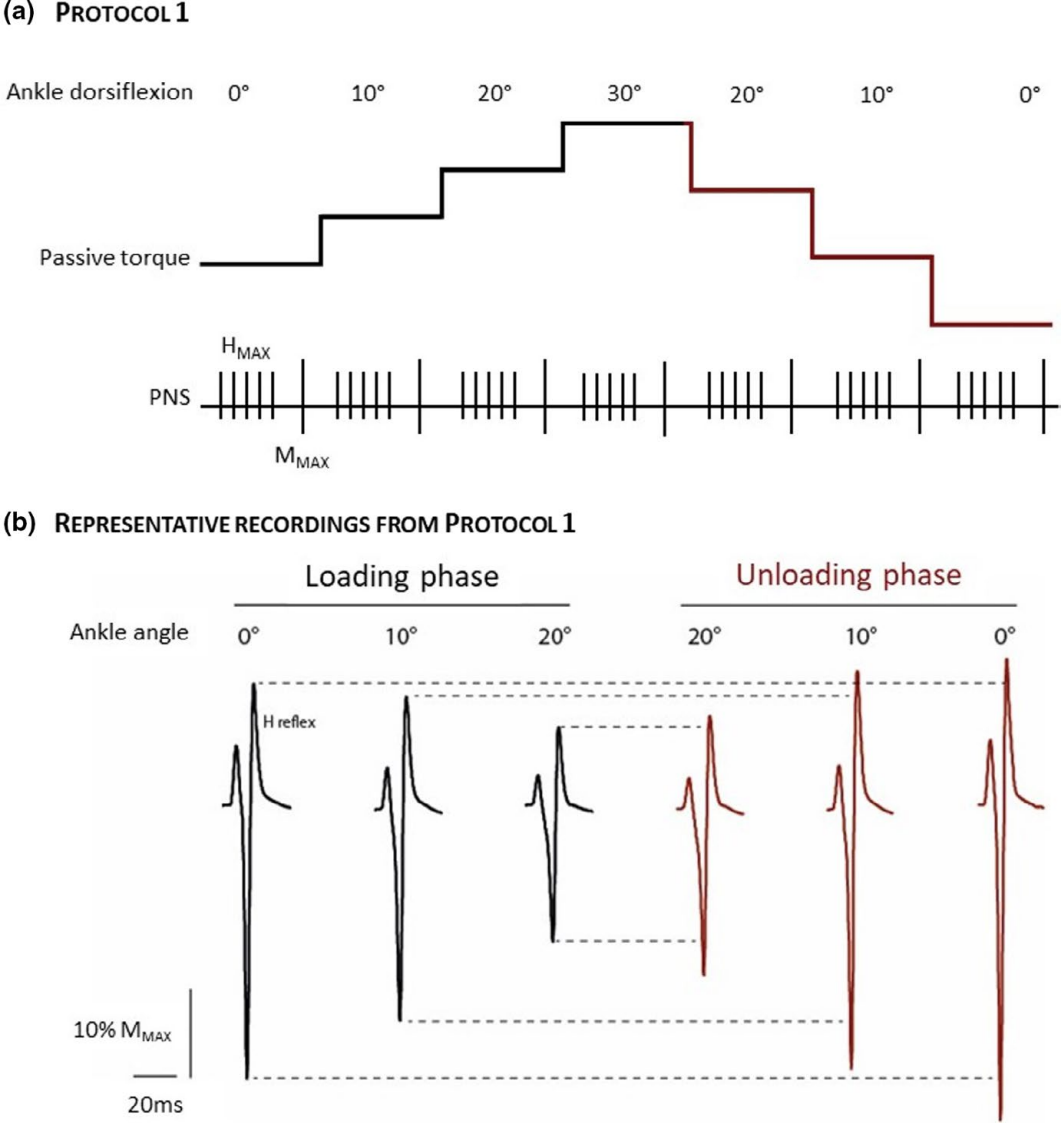
Schematic representation of protocol 1 (a) and representative recordings (averaged over 5 responses) obtained from one participant during protocol 1 (b). The reduced passive torque during the unloading phase in the schematic representation of the protocol illustrates the decline in passive torque during experiments. *H*_max_ and *M*_max_, electrical stimulation to evoke maximal H‐reflex amplitude (*H*_max_) and maximal compound muscle action potential; PNS, peripheral nerve stimulation, respectively

In protocol 1 (*n* = 19), the unloading phase was performed by using the same joint angles as those used during the loading phase (0°, 10°, 20°, and 30°; Figure 1). During the unloading phase of protocol 2 (*n* = 19), the joint angles were adjusted to obtain the same PT as those recorded at 0, 10, and 20° during the loading phase. At the end of the loading phase in protocol 3 (*n* = 19), the ankle was held at 30° for 5 min. At the end of the 5 min, the ankle was quickly returned to the reference position before a second loading phase was performed. protocol 4 (*n* = 13) was designed to investigate the HD modulation during the loading and unloading phases at similar ankle angle between phases. The most common way of quantifying HD in humans is to compare the amplitude of two H reflexes evoked at a similar stimulation intensity and within an interval ranging from 0.2 Hz to 10 Hz (Hultborn et al., 1996). Within this range of frequencies, the amplitude of the second H reflex (*H*_2_) is depressed relative to those of the first H reflex (*H*_1_), and the depression is augmented when the interval between the two stimuli is reduced. The amount of HD is quantified by the ratio *H*_2_/*H*_1_ with lower ratio indicating greater HD. However, Rothwell et al. (1986) observed a lesser decrease in the amplitude of a second H reflex relative to those of a first one, for interstimulus interval <8 s, during a voluntary contraction known to increase Ia afferent discharge through alpha‐gamma coactivation (Allen et al., 2008). This observation is interpreted as being the reflect of an increase in on‐going HD, reducing thereby the efficacy of *H*_1_ to depress *H*_2_. As a consequence, the increase in HD can be associated with an increase in *H*_2_/*H*_1_ ratio. Similar interpretation of an occlusion mechanism between the ongoing level of inhibition and the one produced by the conditioning stimulation has been proposed for other H‐reflex methods (Baudry & Duchateau, 2012; Faist et al., 1996). In our experimental conditions, muscle lengthening causes an increase in the background level of Ia afferent discharge (Lennerstrand & Thoden, 1968), which is associated with an increase in HD (Hultborn et al., 1996). The depression evoked by *H*_1_ should therefore be reduced during muscle stretching, leading to a greater *H*_2_/*H*_1_ ratio. Consequently, an increase in *H*_2_/*H*_1_ ratio during muscle stretching was assumed to reflect an increase in HD. In protocol 4, an interval of 0.5 s (2 Hz) was used to obtain a depression large enough to be analyzed during muscle stretching. Four pairs of stimuli were triggered at each ankle angle during the loading and unloading phases. Only pairs for which the *H*_1_ amplitudes did not differ by more than 5% *M*_max_ relative to *H*_1_ recorded at 0° were used for statistical analysis. At least two pairs of H reflexes were included in the analysis for each ankle angle.

Two protocols were performed during the first experimental session and one during the second experimental session to minimize potential biases due to repetition of the stretching maneuvers. The assignment of protocols 1, 2, and 3, and their order within the two experimental sessions were randomly assigned for each participant. protocol 4 was performed in a fourth experiment to be able to perform two stretching maneuvers: one with adjustments in stimulation intensity and one without. However, the decrease in H‐reflex amplitude during the loading phase was too large to accurately assess HD when stimulation intensity was not adjusted; these data are therefore not presented. Furthermore, six participants were not able to complete this session due to schedule issues. At least 48 h separated two successive experimental sessions. Within the first experimental session, the two protocols were performed 30 min apart, a sufficient time to abolish any change in PT induced by the first stretching maneuver (Mizuno et al., 2013).

### 2.6 Additional experiment

Day et al. (2017) showed a strong correlation between fascicle length and the firing rate of muscle spindle afferents from the tibialis anterior during passive MTU lengthening and shortening. A change in fascicle length between the loading and unloading phases could influence the muscle spindle afferents, and thereby the amplitude of the H reflex. This possibility was investigated afterward in 12 participants, who did not participate to the main experiment, in an experiment similar to protocol 1, which was implemented with ultrasound measurements and H‐reflex recordings in SOL and GM. Fascicles in GM were targeted because of the better ultrasound imaging resolution for this superficial muscle compared with the deeper SOL muscle. As fascicles in SOL and GM exhibit similar changes during ankle movements (Sakuma et al., 2012), it was assumed that fascicles recordings in GM should reflect changes in SOL. Longitudinal images of GM were recorded using real‐time B‐mode ultrasonography (ProSound 75, Aloka) with a 6‐cm width linear‐array probe (7.5 MHz, Aloka) coated with a water‐soluble transmission gel to provide acoustic contact. The probe was held by a custom‐made holster strapped to the leg to ensure a constant orientation and pressure of the probe on the skin. A metallic marker, firmly placed between the skin and the ultrasound probe with adhesive tape, was used for measurement purpose to verify that the probe did not move during the recordings (Abellaneda et al., 2009). Changes in muscle fascicle length during the loading and unloading phases were assessed by positioning the probe at ~30% of the distance between the popliteal crease and the center of the medial malleolus, over the mid‐belly of the GM where at least one muscle fascicle was clearly identified. Three ultrasound images were recorded at each ankle angle (0, 10, 20, and 30°) during the loading phase, and at 0, 10, and 20° during the unloading phase, just prior to the H‐reflex recordings. A procedure similar to that described for protocol 1 was used to record *H*_max_ and *M*_max_, and background EMG in SOL and GM.

### 2.7 Data reduction

#### 2.7.1 Main experiment

The peak‐to‐peak amplitudes of H reflexes (*H*_max_, *H*_1_, and *H*_2_) and *M*_max_ were measured from the unrectified EMG signal. *H*_max_, *H*_1_, and *H*_2_ were normalized to the amplitude of the *M*_max_ delivered at the same ankle angle. In protocols 1, 2, and 3, the *H*_max_ amplitude was averaged from the five *H*_max_ delivered at each ankle angle. In protocol 4, the magnitude of HD was assessed by the ratio *H*_2_/*H*_1_ calculated for each pair of stimuli. PT and the rectified EMG of SOL (aEMG) were averaged across 100‐ms epoch preceding each stimulation (Baudry & Duchateau, 2012). The value of PT measured at 0° prior to stretching maneuver was used as the reference value (0 Nm). To assess the reliability of *H*_max_ (protocols 1, 2, and 3) and *M*_max_ (all protocols) at the beginning of each protocol, the intraclass coefficient correlation (ICC) was calculated. A similar procedure was used for the variation in PT during to the first loading phase. The ICC were 0.81, 0.85, and 0.84 for *H*_max_, *M*_max_, and PT, respectively, indicating an excellent reliability (Cicchetti, 1994).

#### 2.7.2 Additional experiment

Data reduction for *H*_max_, *M*_max_, PT, and aEMG were similar that for protocol 1. Muscle fascicle length was defined as a clearly visible fiber bundle lying between the superficial and deep aponeuroses and measured by using a public domain image program (Image J, National Institutes of Health) along the marked fiber bundle. When the end of the fascicle extended off the acquired ultrasound image, the sine law was used to estimate the total length of the fascicle (Abellaneda et al., 2009). Between 1 and 2 fascicles were analyzed per participant, for a total of 18 fascicles.

### 2.8 Statistics

The statistical analysis was performed with JASP software (version 0.13, NL). The Shapiro–Wilk test confirmed that the data fitted a normal distribution. The Geisser–Greenhouse correction was used when the assumption of sphericity was violated. PT, *H*_max_, *H*_1,_ and *M*_max_ amplitude, *H*_2_/*H*_1_ ratio (main experiment), and fascicle length were analyzed by two‐way ANOVA (phase [loading vs. unloading] × angle [0°, 10, 20° for protocols 1, 2, 3, and Additional Experiment, and 0°, 10°, 20°, 30° for protocol 3]) with a Holm post hoc test when appropriate. To document the change in the dependent variables during the loading phase, including the 30° ankle angle position, a one‐way ANOVA (angle) was performed for protocols 1 and 2. In protocols 1, 2, and 3, the best regression model to fit the relation between the amount of change in PT and *H*_max_ was determined by comparing linear (first order polynomial) and non‐linear (one‐phase exponential decay) regression models by means of the extra sum‐of‐square *F*‐test, pooling together data from the loading and unloading phases. The amount of change was calculated as the difference between the values recorded at each angle to those at the reference position (0°) prior to the loading phase. For all comparisons, the alpha level of significance was set at 0.05. Partial eta squared ($\eta_{p}^{2}$) was used to estimate the effect size for the dependent variables. Values are expressed as mean (SD) in the text and tables, and as mean (standard error of the mean) in figures. For each dependent variable, the details of the statistical analysis are provided in Table 1.

## 3 RESULTS

### 3.1 Main experiment

In the four protocols, the *M*_max_ amplitude slightly decreased with the increase in dorsiflexion angle (*p* < 0.001; Table 1) but no statistical differences were observed between phases (*p* > 0.10). aEMG from SOL did not differ across protocols, phases, or angles, indicating that participants remained relaxed during the different procedures (Table 2).

#### 3.1.1 protocol 1

**FIGURE 2:**
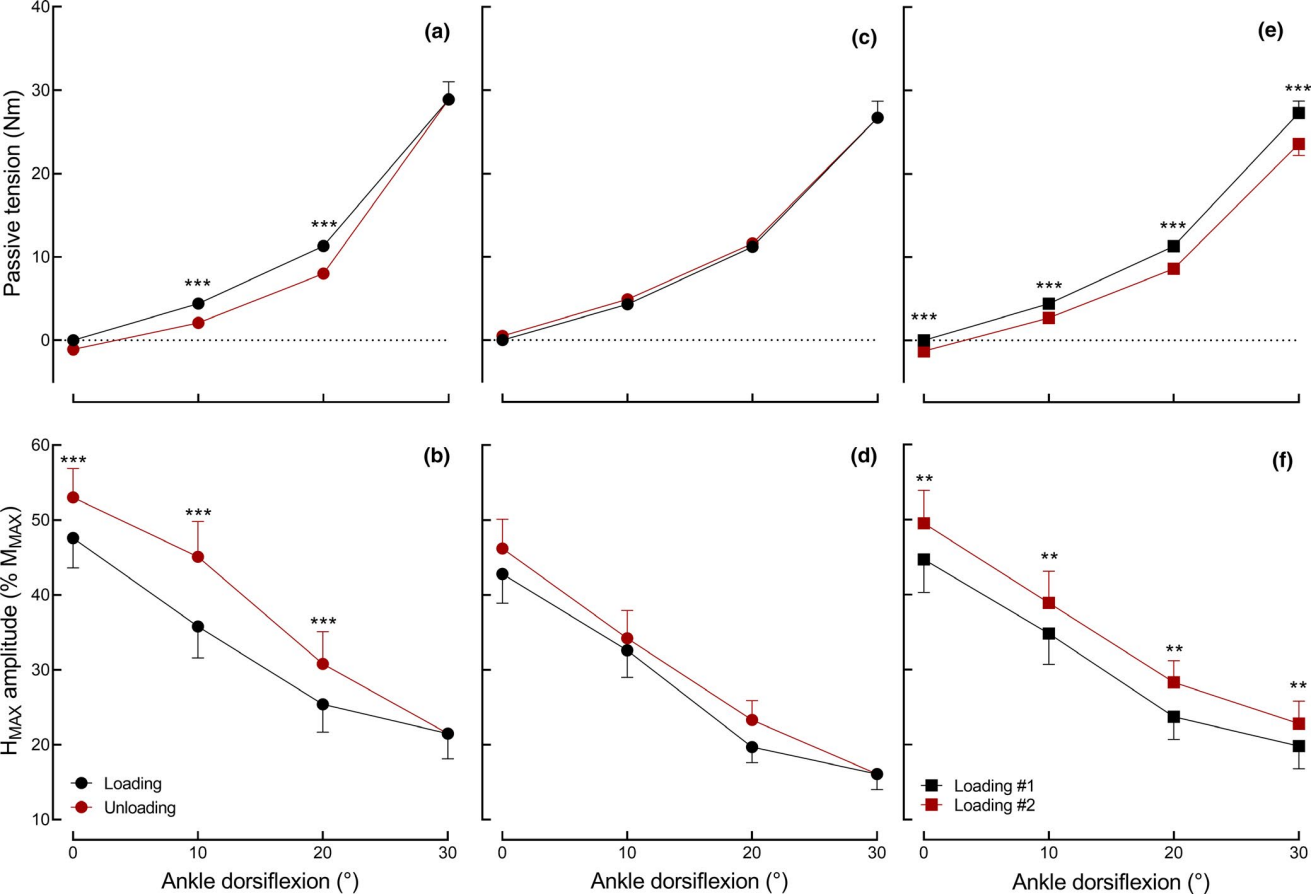
Passive torque and amplitude of the maximal H‐reflex (*H*_max_) during the loading (black dots) and unloading (red dots) phase recorded at similar ankle dorsiflexion angles (protocol 1, a, b), at a constant passive torque (PT) during loading and unloading phases (protocol 2, c, d) and during two loading phases separated by a 5‐min passive hold‐stretch (protocol 3, e, f). The *H*_max_ is expressed as % of the maximal amplitude of the compound muscle action potential (*M*_max_). ** and *** denote a significant difference between phases at *p* < 0.01 and *p* < 0.001, respectively (Holm post‐hoc test). For protocols 1 and 2, only the comparisons between phases at 0, 10, and 20° were provided with statistical symbols (see Methods). Note that for panels (c, d) the ankle angles of the loading phase were used for the *x*‐axis to improve the clarity of the figures. Values are expressed as mean (SE); *n* = 19 (6 women)

**FIGURE 3:**
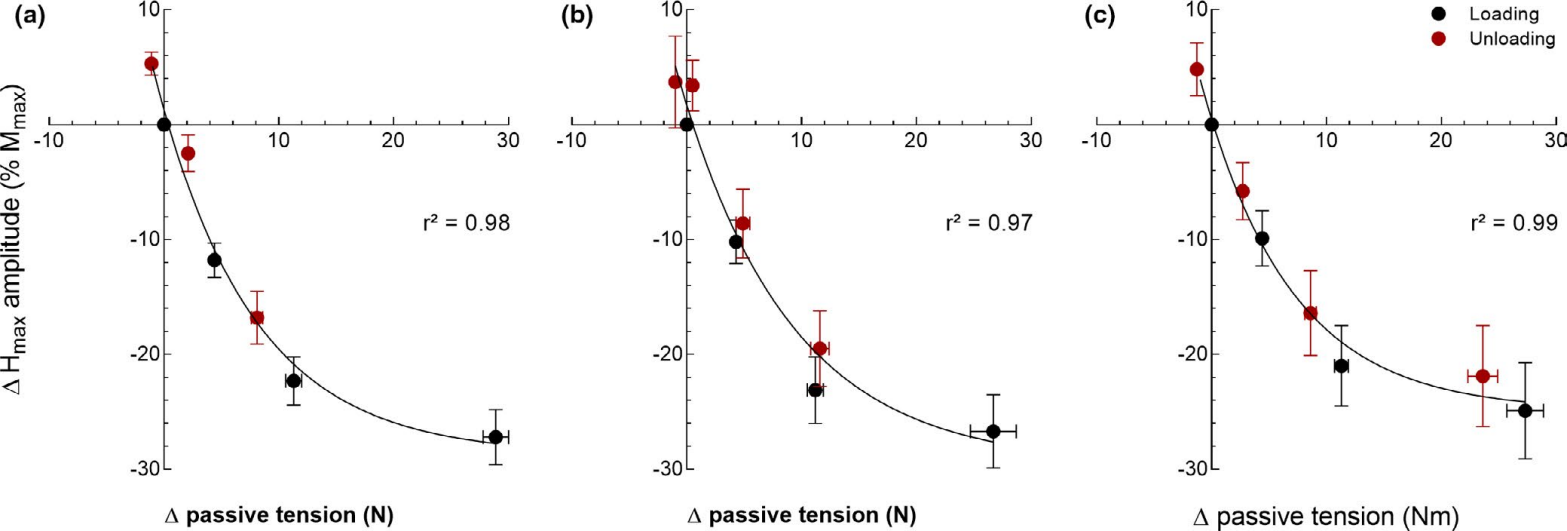
Relation between variations (Δ) in the maximal H‐reflex amplitude (*H*_max_) and passive torque (PT) during the loading (black dots) and unloading (or second loading; protocol 3) (red dots) phases for protocols 1 (a), 2 (b), and 3 (c). Variations are expressed as the difference between the value at each angle and those at 0° prior to the loading phase. The best regression model to fit the data corresponded to a one‐phase exponential decay function with *Y* = 30.0^−0.1.^ *^X^*−28.7 (a), *Y* = 31.3^−0.1.^ *^X^*−29.6 (b) and *Y* = 25.4^−0.1.^ *^X^*−24.9 (c). Values are expressed as mean (SE); *n* = 19 (6 women)

Passive torque increased during the loading phase by 28.9 (8.7) Nm from 0° to 30° ankle angle (*p* < 0.001) (Figure 2a). PT was significantly greater during the loading than unloading phase (*p* < 0.001), with a significant difference between phases for the 10° and 20° ankle angles (*p* < 0.001). The *H*_max_ amplitude decreased during the loading phase from 47.6 (17.4)% *M*_max_ (0°) to 20.4 (15.3)% *M*_max_ (30°) (*p* < 0.001) but was significantly greater during the unloading than the loading phase for each ankle angle (0, 10, and 20°; *p* < 0.001) (Figure 2b). The relation between the amount of change in PT and *H*_max_ amplitude was best fitted (*p* < 0.001) by a one‐phase exponential decay function (*r* ^2^ = 0.98, *Y* = 30.0^−0.1.^ *^X^*−28.7) (Figure 3a).

#### 3.1.2 protocol 2

Passive torque increased by 26.7 (8.5) Nm from 0° to 30° ankle angle during the loading phase (*p* < 0.001) (Figure 2c). PT did not differ between the loading and the unloading phase (*p* = 0.32). The average ankle angle during the unloading phase was 22.3 (1.6)°, 14.4 (1.9)°, and 4.6 (1.2)° to match the PT recorded at 20°, 10°, and 0° ankle angles during the loading phase, respectively. The *H*_max_ amplitude decreased during the loading phase from 42.8 (17.1)% *M*_max_ (0° ankle angle) to 17.8 (10.8)% *M*_max_ (30° ankle angle) (*p* < 0.001) (Figure 2d). There was no statistical difference in *H*_max_ amplitude between phases (*p* = 0.08). The relation between the amount of change in PT and *H*_max_ amplitude was best fitted (*p* < 0.001) by a one‐phase exponential decay function (*r* ^2^ = 0.97, *Y* = 31.3^−0.1.^ *^X^*−29.6) (Figure 3b).

#### 3.1.3 protocol 3

Passive torque increased during the two loading phases (*p* < 0.001) and was significantly reduced during the second loading phase at each ankle angle (0, 10, 20°, and 30°; *p* < 0.001) (Figure 2e). The *H*_max_ amplitude decreased during the two loading phases (*p* < 0.001) but was significantly greater (*p* < 0.001) during the second compared to the first phase (Figure 2f). The relation between the amount of change in PT and *H*_max_ amplitude was best fitted (*p* < 0.001) by a one‐phase exponential decay function (*r* ^2^ = 0.99, *Y* = 25.4^−0.1.^ *^X^*−24.9) (Figure 3c).

#### 3.1.4 protocol 4

**FIGURE 4:**
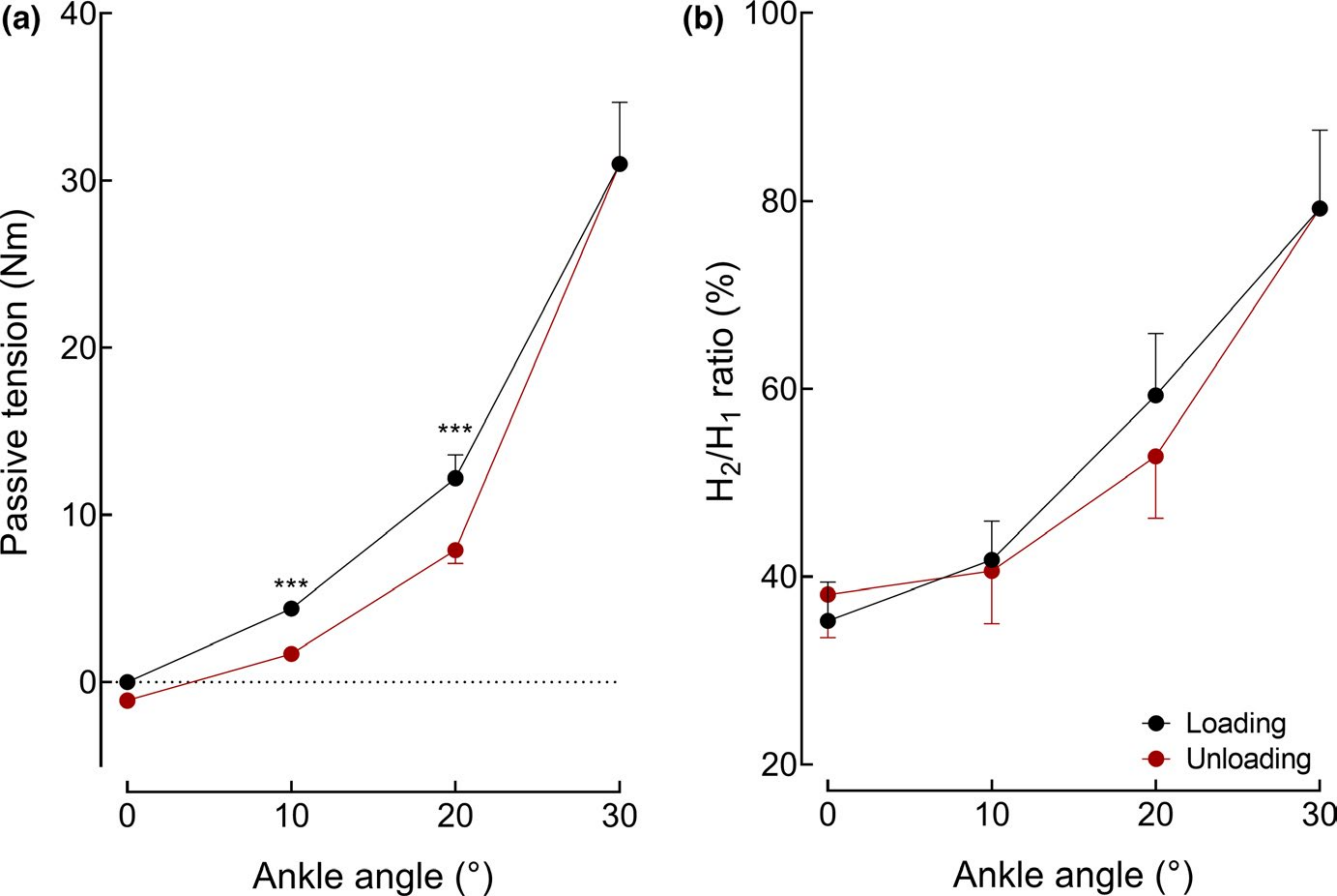
Passive torque (a) and amplitude of the homosynaptic depression (HD, b) during the loading (black dots) and unloading (red dots) phase recorded at similar ankle angles (protocol 4). *** denotes a significant difference between phases at *p* < 0.001 (Holm post‐hoc test). Values are expressed as mean (SE); *n* = 13 (2 women)

Passive torque increased by 31.0 (13.2) Nm from 0° to 30° ankle angle during the loading phase (Figure 4a). PT was significantly lesser during the unloading than loading phase (*p* < 0.001), with a significant difference between phases at the 10° and 20° ankle angles. It was necessary to increase the stimulation intensity during the loading phase and decreased it thereafter during the unloading phase to keep the *H*_1_ amplitude constant. Consequently, the *H*_1_ amplitude did not change during the loading and unloading phases (*p* = 0.69), regardless of the ankle angle (*p* = 0.97, Table 1). The *H*_2_/*H*_1_ ratio increased during the loading phase (*p* = 0.008) and decreased during the unloading phase (Figure 4b). However, there was no statistical difference in *H*_2_/*H*_1_ ratio between phases at a similar ankle angle (*p* = 0.39).

### 3.2 Additional experiment

Passive torque increased during the stretching phase by 31.2 (10.0) Nm from 0° to 30° ankle angle (*p* < 0.001). PT was significantly greater during the loading than unloading phase (*p* < 0.001), with a significant difference between phases for the 10° and 20° ankle angles (*p* < 0.001) (Table 3). The *H*_max_ amplitude in SOL decreased during the loading phase from 51.7 (13.5)% *M*_max_ (0°) to 27.6 (20.2)% *M*_max_ (30°) (*p* < 0.001). The *H*_max_ amplitude was significantly lesser during the loading than unloading phase (*p* < 0.001), with a significant difference at each ankle angle (0, 10, 20°; *p* < 0.001). The *H*_max_ amplitude in GM decreased during the loading phase from 10.6 (6.8)% *M*_max_ (0°) to 5.5 (4.6)% *M*_max_ (30°) (*p* < 0.001), and was lesser during the loading compared to the unloading phase (*p* < 0.001), with a significant difference for each ankle angle (*p* < 0.001) (Table 3). Fascicle length increased during the stretching phase by 12 (5) mm from 0° to 30° ankle angle (*p* < 0.001) but did not differ between the loading and unloading phases (*p* = 0.20). No phase x angle interaction was observed (*p* = 0.49) (Table 3).

**TABLE 1:** *F* values, *p* values, and partial correlations ($\eta_{p}^{2}$) from the ANOVA for the four protocols 1, 2, 3 (*n* = 19, 6 women), and 4 (*n* = 13, 2 women)

	Factors	*F* value	*p* value	$\eta_{p}^{2}$
protocol 1				
PT	**Phase**	**52.6**	**<0.001**	**0.81**
	**Angle**	**92.9**	**<0.001**	**0.89**
	**Phase × angle**	**13.7**	**0.003**	**0.53**
*M*_max_	Phase	<0.1	0.85	<0.01
	**Angle**	**15.0**	**0.001**	**0.56**
	Phase × angle	7.8	0.19	0.13
*H*_max_	**Phase**	**66.9**	**<0.001**	**0.82**
	**Angle**	**92.4**	**<0.001**	**0.92**
	Phase × angle	<0.1	6.2	0.005
aemg	Phase	1.0	0.10	0.34
	Angle	1.7	0.17	0.19
	Phase × angle	1.3	0.13	0.28
protocol 2				
PT	Phase	1.1	0.32	0.06
	**Angle**	**271.5**	**<0.001**	**0.94**
	Phase × angle	0.7	0.49	0.04
*M*_max_	Phase	3.1	0.10	0.15
	**Angle**	**30.8**	**<0.001**	**0.65**
	Phase × angle	0.1	0.882	<0.01
*H*_max_	Phase	3.4	0.08	0.16
	**Angle**	**61.3**	**<0.001**	**0.77**
	Phase × angle	0.6	0.53	0.03
aemg	Phase	0.8	0.40	0.04
	Angle	1.4	0.25	0.07
	Phase × angle	0.4	0.58	0.02
protocol 3				
PT	**Phase**	**78.2**	**<0.001**	**0.83**
	**Angle**	**247.9**	**<0.001**	**0.93**
	**Phase × angle**	**33.6**	**<0.001**	**0.68**
*M*_max_	Phase	0.6	0.46	0.03
	**Angle**	**28.0**	**<0.001**	**0.61**
	Phase × angle	1.0	0.40	0.05
	**Angle**	**29.2**	**<0.001**	**0.62**
	Phase × angle	0.17	0.77	0.01
	Angle	0.4	0.60	0.02
	Phase × angle	0.2	0.80	0.01
protocol 4				
PT	**Phase**	**52.6**	**<0.001**	**0.81**
	**Angle**	**92.9**	**<0.001**	**0.89**
	**Phase × angle**	**13.7**	**0.003**	**0.53**
	**Angle**	**15.0**	**0.001**	**0.56**
	Phase × angle	7.8	0.19	0.13
*H* _1_	Phase	0.2	0.69	0.01
	Angle	0.8	0.44	0.07
	Phase × angle	<0.1	0.97	<0.01
	**Angle**	**7.0**	**0.008**	**0.37**
	Phase × angle	1.9	0.18	0.13
aemg	Phase	0.1	0.71	<0.01
	Angle	1.5	0.24	0.08
	Phase × angle	0.2	0.76	0.01

*n* = 19 for Protocol 1, 2, 3, and *n* = 13 for Protocol 4. Note that for Protocol 3, Phase represents the two loading phases.

Abbreviations: aEMG, averaged value of the rectified EMG; *H* _1_, amplitude of the test H reflex; HD, homosynaptic depression; *M*_max_, maximal amplitude of the M wave; PT, passive torque.

Bold script indicates statistically significant results.

**TABLE 2:** *M*_max_ amplitude (mV) and soleus aEMG (µV) for the four experimental protocols

	Loading phase			Unloading phase^a^		
	0°	10°	20°	0°	10°	20°
*M*_max_						
protocol 1	2.8 (1.0)	2.5 (1.0)	2.3 (0.8)	2.8 (1.0)	2.6 (0.9)	2.3 (0.8)
protocol 2	3.2 (1.0)	2.9 (0.8)	2.6 (0.8)	3.2 (0.9)	2.8 (0.8)	2.5 (0.7)
protocol 3	3.1 (1.1)	2.7 (1.1)	2.4 (0.9)	3.0 (1.2)	2.8 (1.0)	2.4 (0.9)
protocol 4	2.8 (1.2)	2.6 (1.0)	2.4 (0.9)	2.9 (1.2)	2.6 (1.1)	2.3 (1.0)
aEMG						
protocol 1	9.2 (2.8)	9.2 (2.7)	10.0 (5.3)	10.0 (8.3)	10.0 (9.3)	10.0 (5.6)
protocol 2	9.6 (4.5)	10.0 (6.3)	10.0 (4.3)	9.5 (4.2)	10.0 (2.0)	10.0 (6.3)
protocol 3	9.8 (3.6)	9.8 (3.8)	10.0 (3.8)	9.1 (2.3)	8.8 (2.0)	9.1 (2.5)
protocol 4	10.0 (8.3)	10.0 (9.3)	10.0 (5.6)	9.9 (4.5)	9.9 (4.3)	9.1 (2.4)

Abbreviations: aEMG, averaged value of the rectified EMG; *M*_max_, maximal amplitude of the M wave.

^a^ For Protocol 3 = the second loading phase. Data are expressed as mean (SD); Protocols 1, 2, 3 (*n* = 19, 6 women), and 4 (*n* = 13, 2 women).

**TABLE 3:** PT and amplitude of the *H*_max_ and fascicle length recorded in gastrocnemius medialis during the loading and unloading phase during the additional experiment

	Loading phase			Unloading phase		
	0°	10°	20°	0°	10°	20°
PT (Nm)	0 (0)	4.9 (1.6)	14.3 (4.6)	−0.4 (1.1)	3.3 (0.9)**	10.4 (3.2)***
*h*_max_ (%*M*_max_)—SOL	51.7 (13.5)	43.3 (17.5)	33.0 (20.4)	57.2 (15.1)***	52.7 (20.8)***	39.6 (22.6)***
*h*_max_ (%*M*_max_)—GM	10.6 (6.8)	7.6 (4.5)	5.2 (3.6)	12.6 (8.3)***	9.7 (5.7)***	8.7 (6.1)***
Fascicle length (mm)—GM	61.4 (5.1)	67.0 (6.1)	73.4 (9.0)	59.4 (5.2)	66.8 (6.0)	72.1 (4.7)

Values are expressed as mean (SD), *n*=12, 6 women. Values for the 30° ankle angle are not presented as they were not included in phase comparison.

Abbreviations: GM, gastrocnemius medialis; *H*_max_, maximal amplitude of the Hoffmann reflex; PT, passive torque; SOL, soleus.

** and *** = significant difference between phases at *p* < 0.01 and *p* < 0.001 (Holm post‐hoc test), respectively.

## 4 DISCUSSION

The objective of this study was to investigate the influence of PT on the modulation of the SOL H‐reflex pathway during the loading and unloading phases of a passive stretching of the plantar flexor muscles. The results indicate a clear association between PT and the H reflex, such that the lesser the PT, the greater the H‐reflex amplitude. The present results suggest that the inhibitory processes acting on the H‐reflex pathway during muscle stretching are, in part, related to PT developed by the plantar flexor muscles. Potential mechanisms linking PT to H‐reflex modulation follow.

### 4.1 PT during passive stretching

Consistent with previous in vivo studies, PT produced by the plantar flexor muscles increased during passive ankle dorsiflexion (Abellaneda et al., 2009; Guissard & Duchateau, 2004; Morse et al., 2008). The increase in PT during the loading phase results from the forcibly lengthening of muscle ultrastructures (titin, connectin) and connective tissues of the MTU (Bojsen‐Møller et al., 2006; Prado et al., 2005). Furthermore, PT was significantly lower during the unloading than loading phase, illustrating the elastic hysteresis of the MTU (Nordez et al., 2008; Taylor et al., 1990). Due to the 30‐s hold at each ankle angle, the lesser PT during the unloading phase likely resulted also from the stress relaxation mechanism in our experimental conditions. Regardless of the protocol, no EMG activity was recorded during the four protocols, indicating that change in PT mainly reflects a decrease in MTU stiffness (Kay & Blazevich, 2009; Konrad et al., 2017).

### 4.2 Relation between PT and H‐reflex modulation during passive stretching

In agreement with previous work (Guissard et al., 1988; Pinniger et al., 2001), the passive lengthening of the MTU was accompanied by a decrease in *H*_max_ amplitude. In contrast with the well‐documented decrease in *H*_max_ amplitude during the loading phase, no data were available on the modulation of the H reflex during the unloading phase of a stretching maneuver. We observed a greater H‐reflex amplitude during the unloading than loading phase (protocol 1) for similar ankle angle. These results could reflect a relation between PT and the modulation of the H‐reflex pathway during muscle stretching (Guissard & Duchateau, 2006). To confirm such a relation, PT was matched during the loading and unloading phases (protocol 2), with the hypothesis that if the H‐reflex modulation depends in part on PT, a similar PT between phases should minimize differences in the H‐reflex amplitude between phases. In agreement, results from protocol 2 did not show differences in *H*_max_ amplitude between the loading and unloading phases. Finally, to document that the role played by PT on H‐reflex modulation was not only related to the elastic hysteresis, we assessed the effect of reducing MTU stiffness prior to the loading phase with the rationale that lesser PT during the loading phase should be accompanied by greater *H*_max_ amplitude (protocol 3). The results indicate that reducing the increase in PT during the loading phase was accompanied by greater H‐reflex amplitude, supporting therefore our hypothesis. This rationale is further supported by the strong association (*r* ^2^ ≥ 0.97) between the changes in PT and *H*_max_ amplitude in each experimental condition (Figure 3). These results show for the first time a clear influence of PT on the modulation of the H‐reflex pathway during MTU passive stretching.

### 4.3 HD during the unloading phase

Homosynaptic depression, which reflects the decrease in neurotransmitter release by Ia afferent terminals during repetitive activation (Hultborn et al., 1996), is assumed to be partially responsible for the decrease in H‐reflex amplitude during passive MTU lengthening (Cooper, 1961; Hultborn et al., 1996). Recently, Budini et al. (2018) suggested that a reduced HD may contribute to the increase in H‐reflex amplitude during the hold phase of a ramp‐and‐hold stretch. The authors hypothesized that, when plantar flexor muscles are held stretched at a constant ankle angle, the discharge rate of Ia afferents should decrease due to stress relaxation (Cooper, 1961), offering the possibility for neurotransmitters to be restored. In support of this assumption, a decrease in Ia afferent discharge rate was observed in cat musculature during ramp‐and‐hold stretch (Lennerstrand & Thoden, 1968). The lesser PT during the unloading phase could therefore reduce the amount of HD.

In our experiment, the *H*_2_/*H*_1_ ratio increased during the loading phase. The theoretical background of the paired H reflexes suggests that such an increase should reflect a decrease in HD (Hultborn et al., 1996). However, muscle stretching causes an increase in the background level of Ia afferent discharge (Lennerstrand & Thoden, 1968), which should increase the ongoing level of HD (See the Main experiment section). The depression evoked by *H*_1_ would therefore be reduced, leading to a greater *H*_2_/*H*_1_ ratio compared with the reference joint position. As a consequence, an increase in *H*_2_/*H*_1_ ratio during the loading and unloading phases is assumed to reflect an increase in HD. Similar interpretation of a paradoxal change in conditioned H reflex/test H reflex ratio have been proposed for other H‐reflex methods in various experimental conditions (Baudry & Duchateau, 2012; Faist et al., 1996).

During the unloading phase, the *H*_2_/*H*_1_ ratio decreased (less HD) but was similar compared with the loading phase for similar ankle angles. As the amplitude of *H*_1_ was kept constant throughout the stretching procedure, the influence of a change in *H*_1_ amplitude on the amount of change in *H*_2_/*H*_1_ ratio can be eliminated. Overall, these results indicate that HD should contribute to the modulation of the H‐reflex pathway during stretching maneuvers but not in the phase‐dependent modulation of the H‐reflex pathway observed in the present experiment.

### 4.4 Fascicle length behavior

To document a possible role of fascicle length in the phase‐dependent modulation of the H‐reflex pathway, we recorded fascicle length during the loading and unloading phase. Previous work reports a strong correlation between fascicle length and muscle spindle afferent discharge during passive MTU lengthening and shortening (Day et al., 2017). In addition, a decrease in PT across repetitive stretching maneuvers was accompanied by a reduced muscle fascicle lengthening during the stretch (Kato et al., 2011). Accordingly, the lesser PT in the unloading phase could be accompanied by a decrease in fascicle length that may influence the H‐reflex pathway (Additional Experiment). However, even though fascicle length increased and decreased during the loading and unloading phases in GM, respectively, fascicle length did not differ between phases. In contrast, *H*_max_ in GM and SOL was greater in the unloading than the loading phase. Therefore, the greater GM H‐reflex amplitude recorded during the unloading phase was not due to differences in fascicle length between phases. As fascicles in SOL and GM exhibit similar changes during ankle movements (Sakuma et al., 2012), these results can be extended to the modulation of SOL H‐reflex reported in the main experiment. Although ultrasound image may have some limitations in spatial resolution to measure very small fascicle length variations, these additional data suggest that the phase‐dependent modulation of the H reflex did not result from difference in fascicle length between phases.

### 4.5 Other possible mechanism modulating H‐reflex pathway with PT

Guissard and Duchateau (2006) suggested an increase in non‐reciprocal group I inhibition, which is conveyed through Ib afferents originating in Golgi tendon organs (GTO), during large amplitude stretching maneuvers. The GTO were thought to be mainly sensitive to active tension (Houk & Henneman, 1967), until it was shown, in cats, to be also sensitive to low passive forces (Binder et al., 1977; Gregory et al., 2002; Stuart et al., 1970). A decrease in stiffness of the muscular portion of the MTU (Kay & Blazevich, 2009; Konrad et al., 2017) could therefore reduce the straightening of the collagen fibers surrounding the GTO, and thereby reduce the strength of non‐reciprocal group I inhibition. In agreement, Gregory et al. (2002) showed, in cat, that the decrease in passive tension during the unloading phase of a stretch was accompanied by a lower Ib discharge rate in the homonymous muscle (see their Figure 2a). If transferable to humans, this can fit with the greater *H*_max_ amplitude during the unloading phase (protocol 1) and when PT was depressed prior to the loading phase (protocol 3).

Furthermore, presynaptic inhibition of Ia afferents by primary afferent depolarization (PAD) interneurons (Rudomin & Schmidt, 1999) could be involved in the modulation of the H‐reflex pathway for moderate stretch magnitudes (Guissard & Duchateau, 2006). An increase in Ia presynaptic inhibition during the loading phase could reduce the efficacy of Ia afferents to discharge motor neurons during the loading phase. During the unloading phase, an opposite change should occur, with a decrease in Ia presynaptic inhibition. In the cat, Ib afferents converge onto PAD interneurons responsible for Ia presynaptic inhibition (Eccles et al., 1962). A decrease in Ib afferent activity during the unloading phase could therefore reduce the strength of Ia presynaptic inhibition. Although further work is necessary to determine the different mechanisms involved, our results suggest non‐reciprocal group I inhibition as a possible candidate for the relation between PT and the H‐reflex pathway during MTU stretching.

### 4.6 Methodological considerations

A few methodological aspects of this study should be discussed. First, each ankle angle was held for about 30 s, likely allowing for the adaptation of Ia afferent discharge both during the loading and unloading phases (Cooper, 1961; Nielsen et al., 1993). This duration likely diminished the difference in the discharge rate of Ia afferents between the two phases due to the stress‐relaxation phenomenon. However, it was not possible to shorten the duration of each step because of the need to record multiple H reflexes. Therefore, the present results may be more pronounced for briefer step periods during the loading and unloading phases. Second, the *M*_max_ amplitude decreased slightly during the stretching procedure, likely reflecting changes in the muscle geometry (Vigotsky et al., 2018). The *M*_max_ was used to normalize the H reflex amplitude, taking into account this kind of change in recording condition. However, no difference was observed between phases. Accordingly, the phase‐dependent modulation of the H reflex should not be due to changes in recording conditions between phases. Finally, the recording of the complete H‐reflex recruitment curve could have provided more information on the gain modulation of the H‐reflex pathway during the stretching maneuver. However, this would have increased the duration of the maintenance phases at each joint angle too much. The needed adjustments in stimulation intensity for keeping similar the amplitude of *H* _50_ in protocol 4, nonetheless, confirm that the modulation observed in *H*_max_ was also present for submaximal H reflexes.

## 5 CONCLUSION

Our study provides strong evidence that part of the modulation of the H‐reflex pathway during MTU stretching depends on PT, such that lower the PT, greater the H‐reflex. Inhibitory mechanisms originating from Ib afferents could contribute to such a relation between PT and H‐reflex pathway. This study sheds novel insight on the relations between muscle mechanics and neural adjustments during MTU stretching.

## CONFLICT OF INTEREST

None.

## AUTHOR CONTRIBUTIONS

MD, NG, and SB: Conception or design of the work; MD, HEK, CD, NG, and SB: Acquisition and analysis or interpretation of data for the work; MD, NG, and SB: Drafting the work or revising it critically for important intellectual content.

## References

[phy214834-bib-0001] Abellaneda, S. , Guissard, N. , & Duchateau, J. (2009). The relative lengthening of the myotendinous structures in the medial gastrocnemius during passive stretching differs among individuals. Journal of Applied Physiology, 106, 169–177. 18988765 10.1152/japplphysiol.90577.2008

[phy214834-bib-0002] Allen, T. J. , Ansems, G. E. , & Proske, U. (2008). Evidence from proprioception of fusimotor coactivation during voluntary contractions in humans. Experimental Physiology, 93, 391–398. 18039976 10.1113/expphysiol.2007.040741

[phy214834-bib-0003] Baudry, S. , & Duchateau, J. (2012). Age‐related influence of vision and proprioception on Ia presynaptic inhibition in soleus muscle during upright stance. The Journal of Physiology, 590, 5541–5554. 22946095 10.1113/jphysiol.2012.228932 PMC3515837

[phy214834-bib-0004] Behm, D. G. , Blazevich, A. J. , Kay, A. D. , & McHugh, M. (2016). Acute effects of muscle stretching on physical performance, range of motion, and injury incidence in healthy active individuals: A systematic review. Applied Physiology, Nutrition, and Metabolism, 41, 1–11. 10.1139/apnm-2015-0235 26642915

[phy214834-bib-0005] Binder, M. D. , Kroin, J. S. , Moore, G. P. , & Stuart, D. G. (1977). The response of Golgi tendon organs to single motor unit contractions. The Journal of Physiology, 271, 337–349. 925987 10.1113/jphysiol.1977.sp012003 PMC1353575

[phy214834-bib-0006] Bojsen‐Møller, J. , Brogaard, K. , Have, M. J. , Stryger, H. P. , Kjaer, M. , Aagaard, P. , & Magnusson, S. P. (2006). Passive knee joint range of motion is unrelated to the mechanical properties of the patellar tendon. Scandinavian Journal of Medicine and Science in Sports, 17, 415–421. 10.1111/j.1600-0838.2006.00591.x. 17076834

[phy214834-bib-0007] Budini, F. , Christova, M. , Gallasch, E. , Rafolt, D. , & Tilp, M. (2018). Soleus H‐reflex inhibition decreases during 30 s static stretching of plantar flexors, showing two recovery steps. Frontiers in Physiology, 9, 935. 30061844 10.3389/fphys.2018.00935 PMC6054967

[phy214834-bib-0008] Budini, F. , Gallasch, E. , Christova, M. , Rafolt, D. , Rauscher, A. B. , & Tilp, M. (2017). One minute static stretch of plantar flexors transiently increases H reflex excitability and exerts no effect on corticospinal pathways: Spinal and supraspinal responses to muscle stretching. Experimental Physiology, 102, 901–910. 28585766 10.1113/EP086374

[phy214834-bib-0009] Cicchetti, D. V. (1994). Multiple comparison methods: Establishing guidelines for their valid application in neuropsychological research. Journal of Clinical and Experimental Neuropsychology, 16, 155–161. 8150886 10.1080/01688639408402625

[phy214834-bib-0010] Cooper, S. (1961). The responses of the primary and secondary endings of muscle spindles with intact motor innervation during applied stretch. Quarterly Journal of Experimental Physiology and Cognate Medical Sciences, 46, 389–398. 13881160 10.1113/expphysiol.1961.sp001558

[phy214834-bib-0011] Day, J. , Bent, L. R. , Birznieks, I. , Macefield, V. G. , & Cresswell, A. G. (2017). Muscle spindles in human tibialis anterior encode muscle fascicle length changes. Journal of Neurophysiology, 117, 1489–1498. 28077660 10.1152/jn.00374.2016 PMC5376612

[phy214834-bib-0012] Duong, B. , Low, M. , Moseley, A. M. , Lee, R. Y. W. , & Herbert, R. D. (2001). Time course of stress relaxation and recovery in human ankles. Clinical Biomechanics, 16, 601–607. 11470302 10.1016/s0268-0033(01)00043-2

[phy214834-bib-0013] Eccles, J. C. , Schmidt, R. F. , & Willis, W. D. (1962). Presynaptic inhibition of the spinal monosynaptic reflex pathway. The Journal of Physiology, 161, 282–297. 13889059 10.1113/jphysiol.1962.sp006886 PMC1359623

[phy214834-bib-0014] Faist, M. , Dietz, V. , & Pierrot‐Deseilligny, E. (1996). Modulation, probably presynaptic in origin, of monosynaptic Ia excitation during human gait. Experimental Brain Research, 109, 441–449. 8817274 10.1007/BF00229628

[phy214834-bib-0015] Frigon, A. , Carroll, T. J. , Jones, K. E. , Zehr, E. P. , & Collins, D. F. (2007). Ankle position and voluntary contraction alter maximal M waves in soleus and tibialis anterior. Muscle and Nerve, 35, 756–766. 17295303 10.1002/mus.20747 PMC5005069

[phy214834-bib-0016] Giuliani, H. K. , Gerstner, G. R. , Mota, J. A. , & Ryan, E. D. (2019). Age‐related changes in the passive properties of the plantarflexors: Influence of tissue size and quality. Clinical Biomechanics, 68, 53–57. 31158590 10.1016/j.clinbiomech.2019.05.027

[phy214834-bib-0017] Gregory, J. E. , Brockett, C. L. , Morgan, D. L. , Whitehead, N. P. , & Proske, U. (2002). Effect of eccentric muscle contractions on Golgi tendon organ responses to passive and active tension in the cat. The Journal of Physiology, 538, 209–218. 11773329 10.1113/jphysiol.2001.012785 PMC2290032

[phy214834-bib-0018] Guissard, N. , & Duchateau, J. (2004). Effect of static stretch training on neural and mechanical properties of the human plantar‐flexor muscles. Muscle & Nerve, 29, 248–255. 14755490 10.1002/mus.10549

[phy214834-bib-0019] Guissard, N. , & Duchateau, J. (2006). Neural aspects of muscle stretching. Exercise and Sport Sciences Reviews, 34, 154–158. 17031252 10.1249/01.jes.0000240023.30373.eb

[phy214834-bib-0020] Guissard, N. , Duchateau, J. , & Hainaut, K. (1988). Muscle stretching and motoneuron excitability. European Journal of Applied Physiology and Occupational Physiology, 58, 47–52. 3203674 10.1007/BF00636602

[phy214834-bib-0021] Houk, J. , & Henneman, E. (1967). Responses of Golgi tendon organs to active contractions of the soleus muscle of the cat. Journal of Neurophysiology, 30, 466–481. 6037588 10.1152/jn.1967.30.3.466

[phy214834-bib-0022] Hultborn, H. , Illert, M. , Nielsen, J. , Paul, A. , Ballegaard, M. , & Wiese, H. (1996). On the mechanism of the post‐activation depression of the H‐reflex in human subjects. Experimental Brain Research, 108, 450–462. 8801125 10.1007/BF00227268

[phy214834-bib-0023] Kato, E. , Vieillevoye, S. , Balestra, C. , Guissard, N. , & Duchateau, J. (2011). Acute effect of muscle stretching on the steadiness of sustained submaximal contractions of the plantar flexor muscles. Journal of Applied Physiology, 110, 407–415. 21127213 10.1152/japplphysiol.01087.2010

[phy214834-bib-0024] Kawakami, Y. , Kanehisa, H. , & Fukunaga, T. (2008). The relationship between passive ankle plantar flexion joint torque and gastrocnemius muscle and Achilles tendon stiffness: Implications for flexibility. Journal of Orthopaedic & Sports Physical Therapy, 38, 269–276. 10.2519/jospt.2008.2632 18448880

[phy214834-bib-0025] Kay, A. D. , & Blazevich, A. J. (2009). Moderate‐duration static stretch reduces active and passive plantar flexor moment but not Achilles tendon stiffness or active muscle length. Journal of Applied Physiology, 106, 1249–1256. 19179644 10.1152/japplphysiol.91476.2008

[phy214834-bib-0026] King, S. L. , Vanicek, N. , & O'brien, T. D. (2016). Gastrocnemius muscle architecture and Achilles tendon properties influence walking distance in claudicants with peripheral arterial disease: Gastronemius muscle and Achilles tendon in Pad‐IC. Muscle & Nerve, 53, 733–741. 26427003 10.1002/mus.24925

[phy214834-bib-0027] Konrad, A. , Budini, F. , & Tilp, M. (2017). Acute effects of constant torque and constant angle stretching on the muscle and tendon tissue properties. European Journal of Applied Physiology, 117, 1649–1656. 28624851 10.1007/s00421-017-3654-5 PMC5506206

[phy214834-bib-0028] Lennerstrand, G. , & Thoden, U. (1968). Muscle spindle responses to concomitant variations in lenght and in fusimotor activation. Acta Physiologica Scandinavica, 74, 153–165. 4235384 10.1111/j.1748-1716.1968.tb04224.x

[phy214834-bib-0029] Lim, J.‐Y. , Choi, S. J. , Widrick, J. J. , Phillips, E. M. , & Frontera, W. R. (2019). Passive force and viscoelastic properties of single fibers in human aging muscles. European Journal of Applied Physiology, 119, 2339–2348. 31468173 10.1007/s00421-019-04221-7

[phy214834-bib-0030] Maganaris, C. N. , & Paul, J. P. (2000). Hysteresis measurements in intact human tendon. Journal of Biomechanics, 33, 1723–1727. 11006400 10.1016/s0021-9290(00)00130-5

[phy214834-bib-0031] Mizuno, T. , Matsumoto, M. , & Umemura, Y. (2013). Viscoelasticity of the muscle‐tendon unit is returned more rapidly than range of motion after stretching: Retention time of the effect after stretching. Scandinavian Journal of Medicine & Science in Sports, 23, 23–30. 21564309 10.1111/j.1600-0838.2011.01329.x

[phy214834-bib-0032] Morse, C. I. , Degens, H. , Seynnes, O. R. , Maganaris, C. N. , & Jones, D. A. (2008). The acute effect of stretching on the passive stiffness of the human gastrocnemius muscle tendon unit: Stretching and muscle stiffness. The Journal of Physiology, 586, 97–106. 17884924 10.1113/jphysiol.2007.140434 PMC2375574

[phy214834-bib-0033] Nielsen, J. , Petersen, N. , Ballegaard, M. , Biering‐Sørensen, F. , & Kiehn, O. (1993). H‐reflexes are less depressed following muscle stretch in spastic spinal cord injured patients than in healthy subjects. Experimental Brain Research, 97, 173–176. 8131827 10.1007/BF00228827

[phy214834-bib-0034] Nordez, A. , Casari, P. , & Cornu, C. (2008). Effects of stretching velocity on passive resistance developed by the knee musculo‐articular complex: Contributions of frictional and viscoelastic behaviours. European Journal of Applied Physiology, 103, 243–250. 18297303 10.1007/s00421-008-0695-9

[phy214834-bib-0035] Nordez, A. , Casari, P. , Mariot, J. P. , & Cornu, C. (2009). Modeling of the passive mechanical properties of the musculo‐articular complex: Acute effects of cyclic and static stretching. Journal of Biomechanics, 42, 767–773. 19264311 10.1016/j.jbiomech.2008.12.019

[phy214834-bib-0036] Pinniger, G. J. , Nordlund, M. M. , Steele, J. R. , & Cresswell, A. G. (2001). H‐reflex modulation during passive lengthening and shortening of the human triceps surae. The Journal of Physiology, 534, 913–923. 11483720 10.1111/j.1469-7793.2001.00913.x PMC2278740

[phy214834-bib-0037] Prado, L. G. , Makarenko, I. , Andresen, C. , Krüger, M. , Opitz, C. A. , & Linke, W. A. (2005). Isoform diversity of giant proteins in relation to passive and active contractile properties of rabbit skeletal muscles. Journal of General Physiology, 126, 461–480. 10.1085/jgp.200509364 PMC2266601 16230467

[phy214834-bib-0038] Riemann, B. L. , DeMont, R. G. , Ryu, K. , & Lephart, S. M. (2001). The effects of sex, joint angle, and the gastrocnemius muscle on passive ankle joint complex stiffness. Journal of Athletic Training, 36, 369–375. 12937478 PMC155431

[phy214834-bib-0039] Rothwell, J. C. , Day, B. L. , Berardelli, A. , & Marsden, C. D. (1986). Habituation and conditioning of the human long latency stretch reflex. Experimental Brain Research, 63, 197–204. 3732444 10.1007/BF00235664

[phy214834-bib-0040] Rudomin, P. , & Schmidt, R. F. (1999). Presynaptic inhibition in the vertebrate spinal cord revisited. Experimental Brain Research, 129, 1–37. 10550500 10.1007/s002210050933

[phy214834-bib-0041] Sakuma, J. , Kanehisa, H. , Yanai, T. , Fukunaga, T. , & Kawakami, Y. (2012). Fascicle‐tendon behavior of the gastrocnemius and soleus muscles during ankle bending exercise at different movement frequencies. European Journal of Applied Physiology, 112, 887–898. 21687997 10.1007/s00421-011-2032-y

[phy214834-bib-0042] Stuart, D. G. , Goslow, G. E. , Mosher, C. G. , & Reinking, R. M. (1970). Stretch responsiveness of Golgi tendon organs. Experimental Brain Research, 10, 463–476. 4247125 10.1007/BF00234263

[phy214834-bib-0043] Takahashi, R. , Endoh, T. , Nakajima, T. , & Komiyama, T. (2013). Modulation of homosynaptic depression during voluntary contraction and muscle fatigue with different test reflex size. The Journal of Physical Fitness and Sports Medicine, 2, 251–258.

[phy214834-bib-0044] Taylor, D. C. , Dalton, J. D. , Seaber, A. V. , & Garrett, W. E. (1990). Viscoelastic properties of muscle‐tendon units: The biomechanical effects of stretching. The American Journal of Sports Medicine, 18, 300–309. 2372082 10.1177/036354659001800314

[phy214834-bib-0045] Vigotsky, A. D. , Halperin, I. , Lehman, G. J. , Trajano, G. S. , & Vieira, T. M. (2018). Interpreting signal amplitudes in surface electromyography studies in sport and rehabilitation sciences. Frontiers in Physiology, 8, 985. 29354060 10.3389/fphys.2017.00985 PMC5758546

